# A Multicenter Cohort Study on the Association between Metformin Use and Hearing Loss in Patients with Type 2 Diabetes Mellitus Using a Common Data Model

**DOI:** 10.3390/jcm12093145

**Published:** 2023-04-27

**Authors:** Minjin Kim, Dong Heun Park, Hangseok Choi, Insik Song, Kang Hyeon Lim, Hee Soo Yoon, Yoon Chan Rah, June Choi

**Affiliations:** 1 Department of Biostatistics, Korea University College of Medicine, Seoul 02842, Republic of Korea; aron0129@naver.com (M.K.); neuldol@korea.ac.kr (H.C.); 2 Medical Science Research Center, Korea University Ansan Hospital, Ansan 15355, Republic of Korea; 3 Department of Otorhinolaryngology-Head and Neck Surgery, Ansan Hospital, Korea University College of Medicine, Ansan 15355, Republic of Korea; samho502@naver.com (D.H.P.); yhsjoa@hanmail.net (H.S.Y.); ycrah@naver.com (Y.C.R.); 4 Department of Medical Informatics, Korea University College of Medicine, Seoul 02842, Republic of Korea

**Keywords:** common data model, metformin, diabetes mellitus, hearing

## Abstract

We attempted to explore the association between metformin use and hearing loss in in a large-scale study. This retrospective multicenter cohort study assessed the data of patients with type 2 diabetes mellitus (DM) aged over 40 years using the Observational Health Data Science and Informatics open-source software and the Common Data Model database from 1 January 2002 to 31 December 2019. Each participant was selected using the ICD-10-CM diagnosis code E11 for type 2 DM with sensorineural hearing loss. The participants were divided into metformin and non-metformin users. The outcome measure was the first occurrence of hearing loss after the diagnosis of DM as measured by the CDM cohort study. A total of 80,596 patients, including 46,152 metformin users and 34,444 non-metformin users from three hospitals were assessed. After calibration, we compared the risk of hearing loss using Kaplan–Meier curves, and found significant differences between the groups. The calibrated hazard ratio in the three hospitals (0.79 [95% confidence interval, 0.57–1.12]) was summarized. These findings suggest that the probability of hearing loss-free survival in the metformin user group is higher than that in the non-metformin user group.

## 1. Introduction

Hearing loss is defined as the partial or total inability to hear. It can be mild, moderate, or severe, and can affect one or both ears. Currently, approximately 20% of the global population lives with hearing loss [1]. Hearing loss can be congenital, noise-induced, age-related, or caused by ototoxic drug use that damages the inner ear [2].

The impact of hearing loss is broad and can be catastrophic. It includes the loss of the ability to communicate, which can lead to social isolation. Assistive technologies, including hearing aids, cochlear implants, closed captioning, and other devices, can help with hearing loss [3]. However, no definite treatment exists to completely restore the normal hearing system, and only a few prevention techniques, such as hearing aids, are currently available to lower the risk of developing hearing loss.

Noise-induced and age-related hearing losses result from cochlear hair cell injuries that occur through mechanical and metabolic injuries [4]. Commonly accepted mediators that induce cell death include reactive oxygen species (ROS), which cause damage to cells, deoxyribonucleic acid (DNA), proteins, cytosolic molecules, and receptors [5]. To prevent these events, antioxidant substances are being studied for prophylactic use in many hospitals to prevent hearing loss and to configure a prevention system for cochlear hair cell impairment, which can cause damage and metabolic stress as a result of entering the cell death cycle [6].

Metformin, which is commonly administered to patients with diabetes for its antidiabetic effect, is known to have an antioxidant function that blocks the cell apoptosis cycle related to ROS and reduces the oxidative stress levels in rats [7,8]. Several studies have shown that metformin prevents increased cellular ROS production and reduces cell death by regulating intracellular calcium retention and mitochondrial permeability, which leads to reduced DNA damage and mutations [7,9]. Moreover, inner ear cell culture samples have shown that metformin protects against ototoxicity induced by cisplatin and gentamicin by reducing calcium concentration in the cell, reducing the apoptosis rate, and decreasing caspase-3 and poly(adenosine diphosphate-ribose) polymerase production [7,10,11]. Metformin activates adenosine monophosphate-activated protein kinase, which protects the vascular endothelium by participating in signaling cascades that protect the inner ear from damage following overstimulation [10,12,13].

We aimed to validate the relationship between metformin use and hearing loss by using a large-scale common data model (CDM); this relationship has been confirmed in animal experiments and retrospective human studies [8,11]. The CDM cohort study is a correlation analysis of specific topics through a model that defines large-scale clinical data in each hospital with the same standard in structure and definition. Unlike a meta-analysis, CDM may involve: (1) adaptability to specific research; (2) transparency to reproduce findings, assess validity, and inculcate findings; and (3) the ease and speed of utilization [14]. Organizing CDMs preserves the original data from the source and increases its adaptability. Fully organized data models are easy to utilize because all rough codes are mapped to the medical composition. Adaptive rule systems grow a database of reusable measures that can easily adjust to preserve adaptability, expedite analyses, and ensure study-specific transparency [14]. Based on this knowledge, we aimed to compare the risk of hearing loss in patients with type 2 diabetes mellitus (DM) who were either metformin users or non-metformin users.

## 2. Materials and Methods

### 2.1. Study Design and Data Set

This retrospective study of Korean patients who visited the ANAM, GURO, or ANSAN hospital between 1 January 2002 and 31 December 2019 used Observational Health Data Sciences and Informatics (OHDSI) [15] open-source software and the CDM database [16]. The OHDSI network is an international collaboration that aims to develop data-sharing systems [17,18] by applying open-source data analytics to a large number of health databases [19]. This study was approved by the Ethics Committee of all three hospitals (ANAM IRB No. 2022AN0050, GURO IRB No. 2022GR0074, and ANSAN IRB No. 2022AS0006).

### 2.2. Patient Selection

All patients were aged over 40 years, had type 2 DM, and were identified using the ICD-10-CM diagnosis code E11. These are identified by the concept IDs (identifiers) in the CDM database, the details of which are provided in Supplementary Table S1. Among the patients with type 2 DM, we selected those with the first diagnosis of hearing loss using hearing loss-related codes. We excluded patients who had previously been diagnosed with hearing loss and those who had an outcome date within a month from the first date of metformin use. We identified metformin and non-metformin users among the included patients.

### 2.3. Outcomes and Other Variables

The primary outcome of this study was the first occurrence of hearing loss after the diagnosis of type 2 DM. Hearing loss corresponds to sensorineural, sensory, neural, noise-induced, and sudden sensorineural hearing loss, which are mapped to the systematic nomenclature of medicine used as the vocabulary for diagnosis codes in Supplementary Table S2. We excluded the use of aminoglycosides [20,21] and platinum compounds [22,23] while determining covariates because these may militate as confounders of hearing loss regardless of whether metformin is used or not.

We defined potential confounders based on age, sex, history of hyperlipidemia, hypertension, cardiovascular disease, and use of aspirin, nonsteroidal anti-inflammatory drugs, and statins (lovastatin, cilastatin, simvastatin, and rosuvastatin).

### 2.4. Statistical Analysis

This study utilized preference scores, which were obtained by transforming propensity scores (PSs), to measure the relative preference for metformin and non-metformin users in relation to patient demographics and health characteristics. This approach yields scores that are more readily interpretable. The equation for preference scores is as follows: ln((preference score)/(1 − preference score)) = ln((PS)/(1 − PS))-ln(proportion/(1 − proportion)), where “proportion” represents the proportion of participants receiving the treatment [24]. To control for selection bias, we used preference score matching and stratification techniques to balance the covariates between the metformin and non-metformin user groups. In particular, we conducted a matched group analysis using a 1:1 matching ratio with a caliper width of 0.25. In addition, we created a baseline characteristic table to perform Cox proportional regression and calculate the hazard ratio (HR) to assess the association between metformin use and hearing loss. We also used the Kaplan–Meier cumulative survival plot to compare the outcomes before and after employing PS matching and stratification. Furthermore, we conducted a meta-analysis using the data results without calibration, as well as the results obtained after employing matching based on PSs and the effect size of the hospital, and stratification with PSs as correction variables. We evaluated the overall correlations among the variables of interest in the meta-analysis. To test for heterogeneity in the meta-analysis, we used statistical approaches including the Cochran’s Q test and I^2^ value test [25]. Statistical analyses were performed using R version 4.0.3 (http://www.R-project.org accessed on 10 October 2020) and the OHDSI Cohort Method R package [26]. The meta-analyses were conducted using the meta R package with a random-effects model. Although each hospital or organization has its own code of definitions, this can be integrated into the CDM database. For example, in our data, the ANAM, GURO, and ANSAN hospitals might have used slightly different codes for hypertension, but in the CDM database, they used the same codes. Thus, analysis can be performed more easily. In the current study, we demonstrated an association between metformin use and the alleviation of hearing loss among patients.

## 3. Results

### 3.1. Patient Selection and Clinical Characteristics

**Figure 1:**
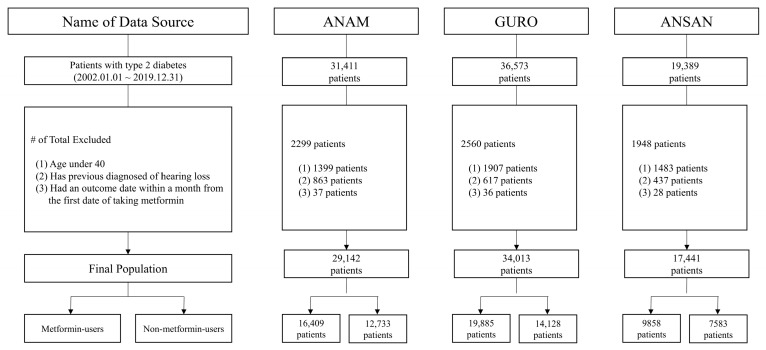
Flowchart of the study participants in the common data model network.

Figure 1 shows the flowchart of the patient selection process. Following exclusion (n = 6807) from 87,373 patients, 80,596 patients from the three hospitals were included in the study (46,152 metformin users and 34,444 non-metformin users). Detailed information about the number of cases and the 1000 person-year incidence rate from each hospital is described in Supplementary Table S3.

The total number of patients from the three hospitals included in the study was 80,596 (46,152 metformin users and 34,444 non-metformin users).

The 1000 person-year incidence rate (IR) of hearing loss in metformin users (IR = 3.64) was slightly higher than that in non-metformin users (IR = 3.58). ANAM hospital showed the same trend (IR = 3.70 to 3.61), whereas the GURO (IR = 3.28 to 3.66) and ANSAN (IR = 4.41 to 4.82) hospitals demonstrated an opposite trend. These results indicated that the time at risk in the metformin group was longer than that in the non-metformin group. In addition, the median follow-up times were 2470.5, 2524.5, and 2062 days for the ANAM, GURO, and ANSAN hospitals, respectively. The follow-up distribution plots are provided in Supplementary Figure S1.

**Figure 2:**
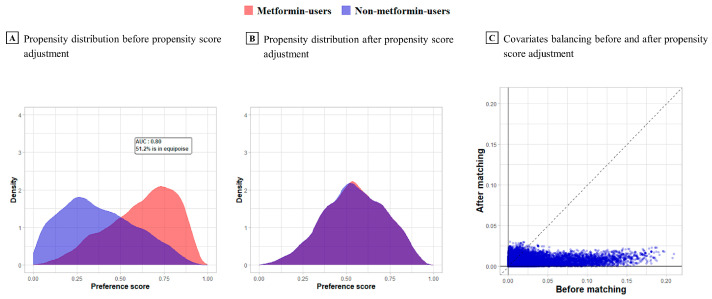
Preference score distribution and covariate balance before and after matching in three hospitals.

Figure 2 presents the preference score distribution, a transformation of PSs adjusted for differences in all hospitals. Figure 2A shows the preference score distribution before matching. The area under the curve (AUC) of the receiver operating characteristic curve for the propensity model can be computed. An AUC of 1 indicates a completely possible treatment assignment based on the baseline covariates, and that the two groups were therefore incomparable [27]. The computed AUC was 0.80 for patient groups from all hospitals. The proportion of overlap according to equipoise was 51.2%. The right side of Figure 2B shows a perfect overlap after PS adjustment, which means that the PS distribution was successful in balancing covariates. Figure 2C shows the covariate balance before and after PS matching. Each dot represents the standardized mean difference for a single covariate before and after PS matching [28]. The X and Y coordinates of each graph show the absolute values of the standardized mean difference before and after PS matching, respectively. Each dot represents a covariate and perfect balance after matching based on most dots in the Y coordinate located below 0.1.

Preference scores are a transformation of PSs adjusted for differences in group size.

The selected baseline characteristics of metformin and non-metformin users from all hospitals before and after PS matching and stratification are described in Table 1 and Table 2, respectively. More than 50% of the patients in both groups were aged between 50 and 74 years. A higher number of metformin users had hypertensive disorder or cardiovascular disease than the number of non-metformin users before PS matching. In addition, the proportion of participants who used medications, such as aspirin and statins, was higher in the metformin user group than that in the non-metformin user group. In contrast, the proportion of participants in the metformin user group who used non-steroidal anti-inflammatory drugs was smaller than that in the non-metformin user group. Moreover, the number of patients who were diagnosed with cardiovascular disease was higher in the non-metformin user group before stratification than that in the non-metformin user group after stratification.

### 3.2. Risk of Hearing Loss Associated with Metformin Use

**Figure 3:**
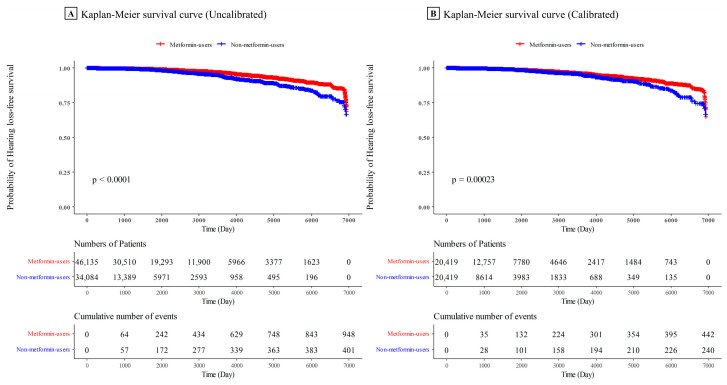
Kaplan–Meier survival curve of hearing loss comparing metformin and non-metformin users.

The current study presents Kaplan–Meier curves for the cumulative risk of hearing loss in patients with diabetes who were metformin or non-metformin users. PS calibration was performed with 10 strata and hospitals (Figure 3). The comparison of each curve showed significant differences in the risk of hearing loss, regardless of calibration. The probability of hearing loss-free survival in metformin users was higher than that in non-metformin users.

The probability of hearing loss-free survival in metformin users was higher than that in non-metformin users.

**Figure 4:**
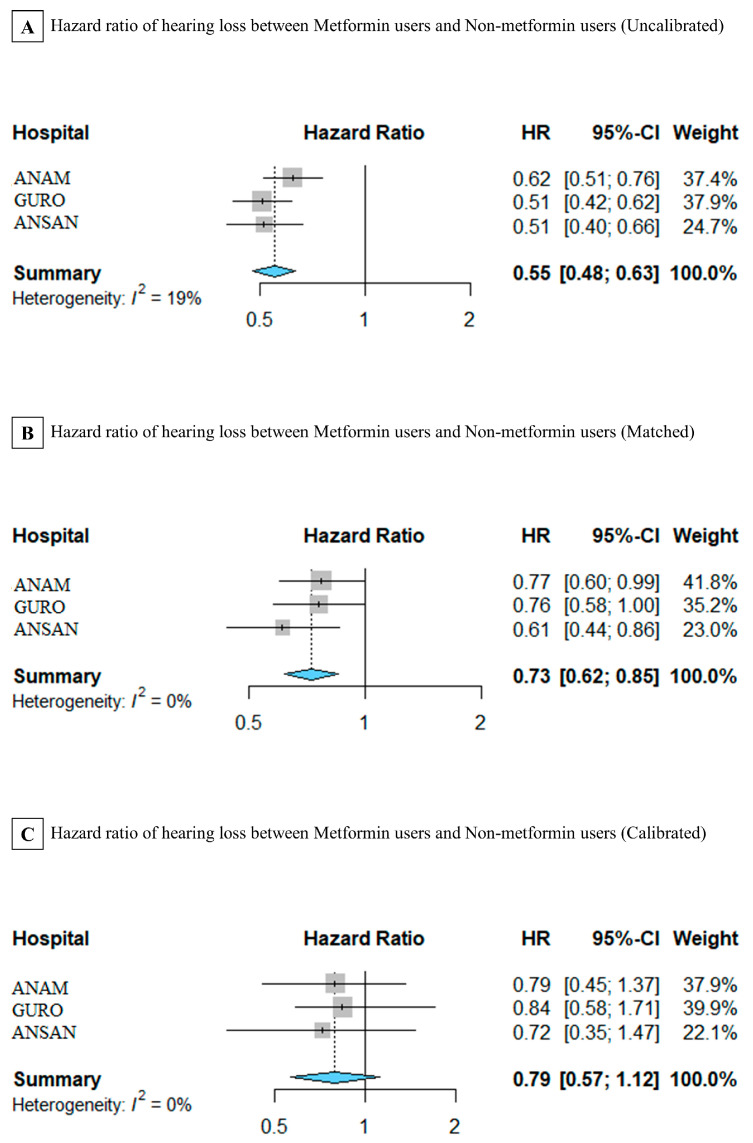
Forest plot of uncalibrated and calibrated hazard ratios (HRs) of metformin use in hearing loss in each hospital. (**A**) HRs are not calibrated by the stratification of propensity scores. The summary was only stratified by institutions. (**B**) HRs are matched by the stratification of propensity scores. The summary includes three hospitals, and (**C**) HRs are calibrated by the stratification of propensity scores and stratified by institutions.

Figure 4 shows the HRs of hearing loss in patients with type 2 DM in the three hospitals. HRs were calibrated by stratification of PSs with 10 strata and stratification of each hospital. Figure 4A shows a forest plot of uncalibrated HRs in the ANAM (HR, 0.62 [95% confidence interval (CI), 0.51–0.76]), GURO (HR, 0.51 [95% CI, 0.42–0.62]), and ANSAN (HR, 0.51 [95% CI, 0.40–0.66]) hospitals and in the summary of the three hospitals (HR, 0.55 [95% CI, 0.40–0.66]). The HRs of hearing loss in metformin users were low, since each HR and CI value was less than 1, which indicated significance. Figure 4B shows a forest plot of HRs in ANAM (HR, 0.77 [95% CI, 0.60–0.99]), GURO (HR, 0.76 [95% CI, 0.58–1.00]), and ANSAN (HR, 0.61 [95% CI, 0.44–0.86]) hospitals after matching, and the summary of the three hospitals (HR, 0.73 [95% CI, 0.62–0.85]). Similarly, the HRs of hearing loss in metformin users were significantly low in patients with DM from the ANAM and ANSAN hospitals, since each HR and CI value was less than 1. Figure 4C shows a forest plot of calibrated HRs in the ANAM (HR, 0.79, [95% CI, 0.45–1.37]), GURO (HR, 0.84 [95% CI, 0.58–1.71]), and ANSAN (HR, 0.72 [95% CI, 0.35–1.47]) hospitals after matching, and the summary of the three hospitals (HR, 0.79 [95% CI, 0.57–1.12]). The comparison of Figure 4A–C showed a slight difference in trend and fair agreement. Calibrated HRs were not significantly different, but they showed a similar trend in Figure 4A,B. Therefore, even calibrated HRs were not significant; a slight difference was found between metformin and non-metformin users. To interpret the result of calibrated HRs in the summary of the three hospitals, the risk of hearing loss in patients with type 2 DM who used metformin was 0.79 times lower than that of patients who did not use metformin over time.

## 4. Discussion

Hearing loss causes difficulty to patients, and they often seek effective treatments; therefore, a drug that could decrease hearing loss would be a great discovery. Among the candidate drugs is metformin, which is known and proven to be safe and is already prescribed to patients with DM worldwide. The mechanism of action of metformin in cochlear hair cells has only been confirmed in animal models through several studies [10,11]; however, conducting a clinical study in humans is challenging. CDM models provide accurate analysis that is possible through the uniformity of large-scale data, and are reproducible. Therefore, we performed the first large-scale CDM cohort study to investigate the association between hearing loss and metformin use in patients with type 2 DM. The CDM database is built on aspects of the data definition system that are accessible to authorized people. Therefore, we performed the first large-scale CDM cohort study to investigate the association between hearing loss and metformin use in patients with type 2 DM.

Based on the data of a large patient group in three hospitals, the groups were homogenized and analyzed according to metformin use. Variations in the patient group were corrected through covariate balancing, PS matching, and stratification. PS indicates the probability that a particular patient is assigned to a treatment group according to the given covariates. These probabilities are calculated using a logistic regression, and each patient has a PS. When we observed differences in the covariates between two groups, we calculated the standard mean difference (SMD). The SMD represents the similarity of the distribution of covariates of both groups. This is known as PS matching.

In this study, the IR of hearing loss for each hospital was higher in the metformin user group, with the exception of the group from the ANSAN hospital. This finding should be considered with exposure time, which is the time at risk of DM; therefore, the metformin users showed a lower incidence rate than non-metformin users. In the demographics of each group, the prevalence of hypertension and cardiovascular disease was higher in metformin users before PS matching and stratification. However, no significant difference was found between the two groups after correction with PS matching and stratification. Furthermore, no significant differences were noted between other potential confounders.

We used a random-effects model to assume the effect size considering the difference in each hospital, whereas the fixed-effects model considered each hospital identically. The most evident difference between the two methods was the variation control. While fixed-effects models are used only within variations, random-effects models are used between variations.

The mechanism underlying the association between metformin use and its risk-reducing effect on hearing loss has not been fully elucidated. In vitro research has suggested that metformin is a potential agent against ototoxicity that reduces the number of ROS produced by all processes [29]. In our clinical cohort study, the probability of hearing loss-free survival in the metformin user group was higher than that in the non-metformin user group, as indicated by the survival and Kaplan–Meier curves after calibration with 10 strata and stratification of the three hospitals. Our data also indicated that among patients with type 2 DM, the HR of hearing loss in metformin users was lower than that in non-metformin users. This finding is consistent with those from studies that reported the anti-inflammatory, neuroprotective, and neuro-regenerative associations of metformin, which have been proven only in animal models [30]. The antioxidative function of metformin blocks ROS production that causes long-term damage to hair cells and prevents the progression of the cell death cycle cascade in clinical outcomes [5]. This function has been confirmed only in animal models but not in humans yet. Further studies on metformin may provide clues to solve the problem of hearing loss, a cause of significant personal and social issues. This study has a few limitations. First, patients were possibly misclassified as having type 2 diabetes by using only ICD-10 codes. Data on the severity of DM (such as hemoglobin A1C level monitoring) were not available, making it difficult to identify and analyze the exact DM severity. Second, information on the precise dose and duration of metformin administration were not available. The analysis of these data is potentially helpful in determining the optimal dosage of metformin required for preventing hearing loss. Third, the database did not provide audiometric results assessing the degree of hearing loss and indicating the prognosis of the population with DM. In addition, because this was a population-based study, we could not clarify the actual mechanism underlying the association between metformin use and hearing loss. Finally, the results of the calibrated HRs were non-significant and were questionable for robustness, and therefore requires a more flexible modeling approach.

Therefore, future randomized prospective studies are warranted to determine the protective effects of metformin on hearing loss in humans. DM medications have the same mechanisms of action as metformin; therefore, disentangling their associations from those of metformin could confirm and redeem the previous results. In addition, in vivo studies are needed to identify and understand the direct mechanism of action of metformin on ear cells. This research will serve as a bridge for the development of innovative therapeutics for the prevention of hearing loss in the future.

## 5. Conclusions

This cohort study using CDM data found that among patients with DM, those who used metformin had a lower risk of hearing loss than those who did not use metformin. These findings suggest an association between hearing loss and metformin use. This pioneering study suggests a new aspect for hearing loss, for which a prominent preventive method has yet to be identified. Several prior experiments in animal models support this finding; however, confirming this in a large-scale patient group by a retrospective cohort study based on the CDM method is necessary. Further research on the mechanism underlying this association and the possible prophylactic methods in patients with hearing loss by complementing these limitations is warranted.

## Figures and Tables

**Table 1:** Selected baseline characteristics of the study participants before and after propensity score matching.

Characteristic	Before Matching			After Matching		
	Metformin Users (n = 46,152)	Non-Metformin Users (n = 34,444)	Standard Difference	Metformin Users (n = 20,419)	Non-Metformin Users (n = 20,419)	Standard Difference
	No. (%)	No. (%)		No. (%)	No. (%)	
Age (years)						
40–44	2641 (5.7)	1298 (3.8)	0.10	945 (4.6)	884 (4.3)	0.01
45–49	3961 (8.6)	2324 (6.8)	0.07	1527 (7.5)	1520 (7.4)	0
50–54	5752 (12.5)	3313 (9.6)	0.09	2102 (10.3)	2158 (10.6)	−0.01
55–59	7143 (15.5)	4422 (12.9)	0.08	2897 (14.2)	2830 (13.9)	0.01
60–64	7337 (15.9)	5140 (14.9)	0.02	3175 (15.5)	3163 (15.5)	0
65–69	6692 (14.5)	5087 (14.8)	−0.01	3036 (14.9)	3071 (15)	−0.01
70–74	5705 (12.4)	5047 (14.7)	−0.06	2920 (14.3)	2888 (14.1)	0.01
75–79	4192 (9.1)	4189 (12.2)	−0.10	2221 (10.9)	2227 (10.9)	0
≥80	2729 (5.9)	3624 (10.5)	−0.32	1596 (7.8)	1677 (8.2)	−0.04
Male	25,967 (56.3)	19,310 (56.1)	0.01	11,452 (56.1)	11,333 (55.5)	0.01
Female	20,185 (43.7)	15,134 (44)	−0.01	8967 (43.9)	9086 (44.5)	−0.01
Hyperlipidemia	5889 (12.8)	2930 (8.5)	0.14	2093 (10.3)	2262 (11.1)	−0.03
Hypertensive disorder	9446 (20.5)	6018 (17.5)	0.10	3785 (18.5)	4011 (19.6)	−0.02
Cardiovascular disease	17,251 (37.4)	11,434 (33.2)	0.15	7128 (34.9)	7419 (36.3)	−0.05
Aspirin	15,832 (34.3)	7969 (23.2)	0.24	5079 (24.9)	5317 (26)	−0.03
Non-steroidal anti-inflammatory drugs	21,982 (47.6)	20,010 (58.2)	−0.21	10,682 (52.3)	10,343 (50.7)	0.03
Statins	15,806 (34.2)	7676 (22.3)	0.20	8026 (39.3)	8126 (39.8)	−0.01

**Table 2:** Selected baseline characteristics of the study participants before and after propensity score stratification.

Characteristic	Before Stratification			After Stratification		
	Metformin Users (n = 46,152)	Non-Metformin Users (n = 34,444)	Standard Difference	Metformin Users (n = 46,152)	Non-Metformin Users (n = 34,399)	Standard Difference
	No. (%)	No. (%)		No. (%)	No. (%)	
Age (years)						
40–44	2641 (5.7)	1298 (2.8)	0.10	2236 (4.8)	1559 (3.4)	0.01
45–49	3961 (8.6)	2324 (5)	0.07	3508 (7.6)	2577 (5.6)	0.00
50–54	5752 (12.5)	3313 (7.2)	0.09	5099 (11)	3617 (7.8)	0.02
55–59	7143 (15.5)	4422 (9.6)	0.08	6639 (14.4)	4824 (10.5)	0.02
60–64	7337 (15.9)	5140 (11.1)	0.02	7143 (15.5)	5402 (11.7)	−0.01
65–69	6692 (14.5)	5087 (11)	−0.01	6756 (14.6)	5060 (11)	0
70–74	5705 (12.4)	5047 (10.9)	−0.06	6308 (13.7)	4757 (10.3)	0
75–79	4192 (9.1)	4189 (9.1)	−0.10	4899 (10.6)	3718 (8.1)	0
≥80	2729 (5.9)	3624 (7.9)	−0.32	3565 (7.7)	2884 (6.2)	−0.06
Male	25,967 (56.3)	19,310 (41.8)	0.01	25,885 (56.1)	19,311 (41.8)	0
Female	20,185 (43.7)	15,134 (32.8)	−0.01	20,267 (43.9)	15,088 (32.7)	0
Hyperlipidemia	5889 (12.8)	2930 (6.3)	0.14	4942 (10.7)	3915 (8.5)	−0.01
Hypertensive disorder	9446 (20.5)	6018 (13)	0.10	8596 (18.6)	6819 (14.8)	−0.01
Cardiovascular disease	17,251 (37.4)	11,434 (24.8)	0.15	7289 (15.8)	5794 (12.6)	−0.01
Aspirin	15,832 (34.3)	7969 (17.3)	0.24	16,018 (34.7)	12,349 (26.8)	−0.02
Non-steroidal anti-inflammatory drugs	21,982 (47.6)	20,010 (43.4)	−0.21	21,529 (46.6)	15,983 (34.6)	0
Statins	15,806 (34.2)	7676 (16.6)	0.20	11,504 (24.9)	8768 (19)	0

## Data Availability

The datasets analyzed during the study are not publicly available due to the legal restrictions of South Korea but are available from the corresponding author on reasonable request.

## Supplementary Materials

The following supporting information can be downloaded at https://www.mdpi.com/article/10.3390/jcm12093145/s1, Supplementary Table S1: List of concept IDs and definitions; Supplementary Table S2: List of ICD-10-CM codes and concept IDs of CDM for hearing loss; Supplementary Table S3: Number of metformin and non-metformin users among the study participants; Supplementary Figure S1: Follow-up distribution plot of each hospital.
