# Supplementary material for: A Multicenter Cohort Study on the Association between Metformin Use and Hearing Loss in Patients with Type 2 Diabetes Mellitus Using a Common Data Model
Source: J Clin Med. 2023 Apr 27;12(9):3145. doi: 10.3390/jcm12093145 (PMC10179543; doi:10.3390/jcm12093145)

Supplementary Information

Supplementary Table S1. List of concept IDs and definitions

Definition	Concept IDs
**Type 2 diabetes mellitus**	201826, 4193704
**Metformin use**	2057647, 2057655, 2057662, 2057670, 2057677, 19106521, 21081251, 21091002, 21091002, 21130197, 21169719, 36887702, 40164885, 40164891, 40164894, 42708090, 42708168, 42708172, 42708176, 42942992, 42953698, 42953740, 42953818, 42953917, 42960587, 42960590, 42960593, 42960642, 42960645, 42960648, 42960759, 42960762, 42960765, 42960771, 42961170, 42961173, 42961319, 42961322, 42961325, 42961484, 42961487, 42961490, 42961494, 43013896, 43013899, 43267262, 45775456, 45775620, 46287408, 46287680, 46287689,
**Hyperlipidemia**	432867, 438720, 4029305, 4120314
**Hypertensive disorder**	316866, 319826, 320128, 4028741, 4071202, 4167493, 4279525, 45768449
**Cardiovascular disorder**	313217, 316139, 317576, 321588, 381591, 4185932
**Aspirin use**	1112807, 1113302, 1113348, 1113350, 1718407, 36211893, 36211894, 36213167, 36213168, 36243877, 40009855, 40009857, 40009890, 40009891, 40009896
**NSAID use**	704943, 920458, 985247, 988294, 991876, 1113648, 1113672, 1115008, 1115125, 1118084, 1124300, 1125315, 1136980, 1146810, 1146847, 1146882, 1150345, 1150871, 1153928, 1156378, 1177480, 1177665, 1178663, 1178665, 1185922, 1189754, 1189760, 1189773, 1195492, 1195496, 1197736, 1201620, 1308738, 1360332, 1395573, 1518254, 1713332, 1717327, 19005046, 19007824, 19011355, 19019050, 19019273, 19019620, 19019979, 19021918, 19022103, 19029024, 19029025, 19029393, 19029394, 19041220, 19049709, 19056874, 19069425, 19069426, 19070224, 19071691, 19071692, 19072152, 19077246, 19078461, 19080243, 19086910, 19086932, 19088915, 19088916, 19091241, 19095309, 19096060, 19101639, 19101640, 19103397, 19103398, 19103697, 19124906, 19133331, 19133853, 19137312, 40057838, 40164851, 42708549, 46287423, 46287424
**Statin use**	922570, 1510813, 1539403, 1549686, 1592085, 1797258, 19136066, 40165636, 43009071
**Aminoglycoside**	902722, 1784749, 1790868, 1836191, 3035509, 3035510, 3036152, 19017585, 21602990, 21602991, 21602994, 45892419
**Platinum compound**	1318011, 1344905, 1397599

NSAID, non-steroidal anti-inflammatory drug; ID,

Supplementary Table S2. List of ICD-10-CM codes and concept IDs of the CDM for hearing loss

ICD-10-CM code	Concept IDs
H91.2	37110393
H91.20A	374053
H90.5E	444291
H90.4	374366
H83.3B	440422
H90.5C	381312
H91.81	374367

CDM, common data model; ICD-10-CM, International Classification of Diseases, Tenth Revision, Clinical Modification; IDs, identifiers.

Supplementary Table S3. Number of metformin and non-metformin users among the study participants

Center	Case	No. of participants*	Hearing loss	Incidence Rate**	Time at Risk
ALL	Metformin-users	46,152	948	3.64	712.60
	Non-metformin-users	34,399	401	3.58	285.22
ANAM hospital	Metformin-users	16,409	364	3.70	272.73
	Non-metformin-users	12,733	151	3.61	114.74
GURO hospital	Metformin-users	19,877	375	3.28	312.96
	Non-metformin-users	13,962	157	3.66	117.64
ANSAN hospital	Metformin-users	9,850	209	4.41	129.80
	Non-metformin-users	7,553	93	4.82	52.85

*ANAM hospital: excluded participants with outcomes prior to the risk window start date (n=5) and without at least 1 day at risk (n=160)
GURO hospital: excluded participants with outcomes prior to the risk window start date (n=4) and without at least 1 day at risk (n=170)
ANSAN hospital: excluded participants with outcomes prior to the risk window start date (n=3) and without at least 1 day at risk (n=35)
**Incidence rates are not adjusted. Incidence rate per 1,000 person-years

Supplementary Figure S1. Follow-up distribution plots of each hospital.

A Follow-up distribution in ANAM hospital

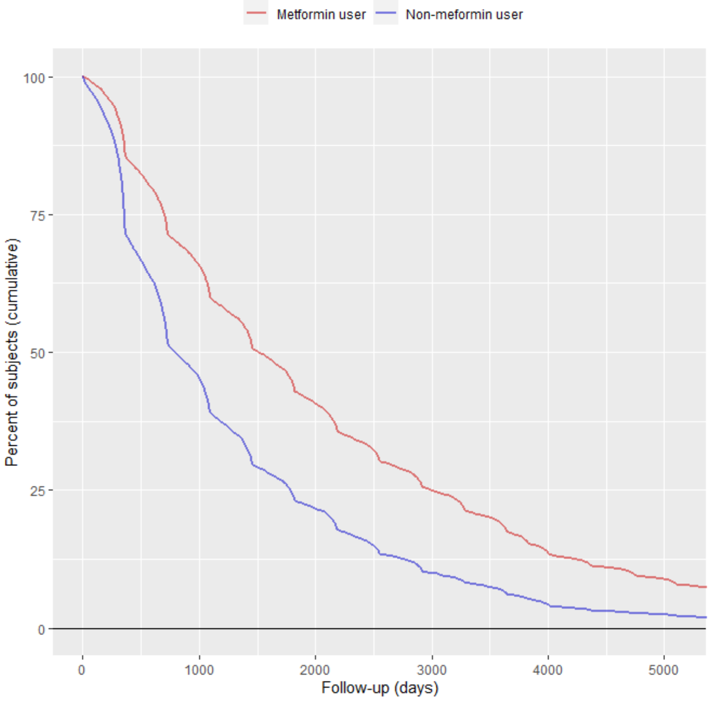

B Follow-up distribution in GURO hospital

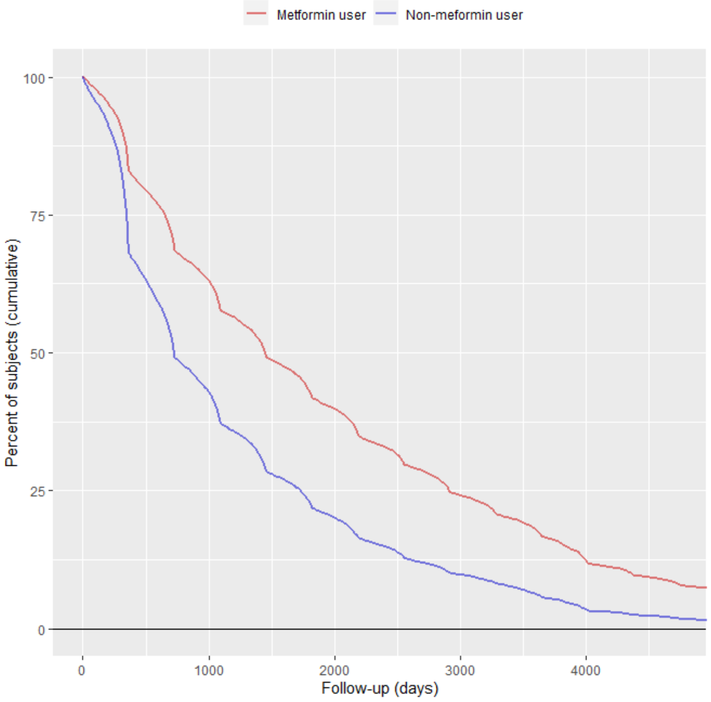

C Follow-up distribution in ANSAN hospital

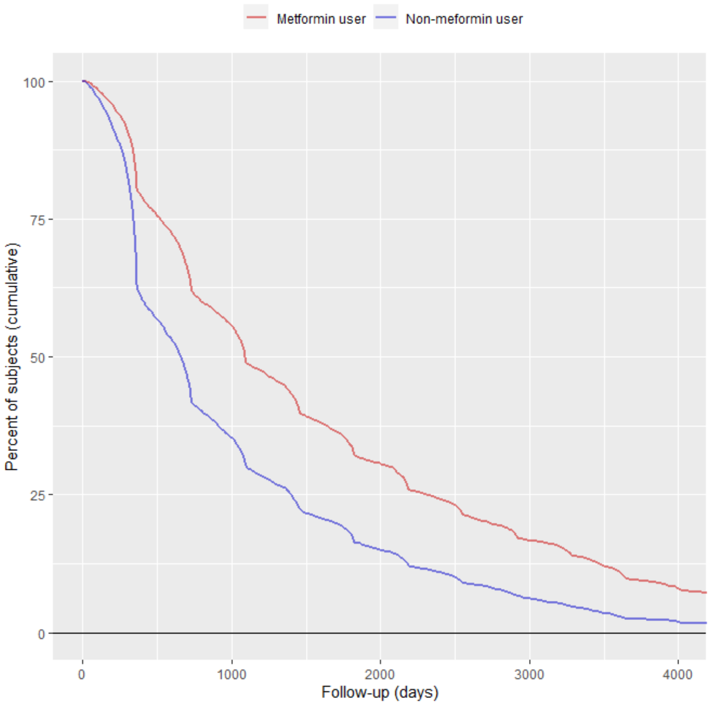

Supplement: Supplementary file 1 [file jcm-12-03145-s001.zip › jcm-2327636-supplementary.pdf]
